# Creation of Tissue-Engineered Urethras for Large Urethral Defect Repair in a Rabbit Experimental Model

**DOI:** 10.3389/fped.2021.691131

**Published:** 2021-06-22

**Authors:** Maria Virginia Amesty, Clara Ibel Chamorro, Pedro López-Pereira, María José Martínez-Urrutia, Beatriz Sanz, Susana Rivas, Roberto Lobato, Magdalena Fossum

**Affiliations:** ^1^Department of Pediatric Urology, Hospital Universitario La Paz, Madrid, Spain; ^2^Department of Women's and Children's Health, Bioclinicum J10:20, Karolinska Institutet, Stockholm, Sweden; ^3^Department of Cell Culture, IdiPAZ Instituto de Investigación Hospital Universitario La Paz, Madrid, Spain; ^4^Division of Pediatric Surgery, Department of Surgical Gastroenterology, Copenhagen University Hospital Rigshospitalet, Copenhagen, Denmark; ^5^Department of Health Sciences, Copenhagen University, Copenhagen, Denmark

**Keywords:** tissue-engineering, regenerative medicine, tissue-engineered urethra, bladder washing, SIS matrix, hypospadias, hypospadias rabbit model, urethroplasty

## Abstract

**Introduction:** Tissue engineering is a potential source of urethral substitutes to treat severe urethral defects. Our aim was to create tissue-engineered urethras by harvesting autologous cells obtained by bladder washes and then using these cells to create a neourethra in a chronic large urethral defect in a rabbit model.

**Methods:** A large urethral defect was first created in male New Zealand rabbits by resecting an elliptic defect (70 mm^2^) in the ventral penile urethra and then letting it settle down as a chronic defect for 5–6 weeks. Urothelial cells were harvested noninvasively by washing the bladder with saline and isolating urothelial cells. Neourethras were created by seeding urothelial cells on a commercially available decellularized intestinal submucosa matrix (Biodesign® Cook-Biotech®). Twenty-two rabbits were divided into three groups. Group-A (*n* = 2) is a control group (urethral defect unrepaired). Group-B (*n* = 10) and group-C (*n* = 10) underwent on-lay urethroplasty, with unseeded matrix (group-B) and urothelial cell-seeded matrix (group-C). Macroscopic appearance, radiology, and histology were assessed.

**Results:** The chronic large urethral defect model was successfully created. Stratified urothelial cultures attached to the matrix were obtained. All group-A rabbits kept the urethral defect size unchanged (70 ± 2.5 mm^2^). All group-B rabbits presented urethroplasty dehiscence, with a median defect of 61 mm^2^ (range 34–70). In group-C, five presented complete correction and five almost total correction with fistula, with a median defect of 0.3 mm^2^ (range 0–12.5), demonstrating a significant better result (*p* = 7.85 × 10^−5^). Urethrography showed more fistulas in group-B (10/10, versus 5/10 in group-C) (*p* = 0.04). No strictures were found in any of the groups. Group-B histology identified the absence of ventral urethra in unrepaired areas, with squamous cell metaplasia in the edges toward the defect. In group-C repaired areas, ventral multilayer urothelium was identified with cells staining for urothelial cell marker cytokeratin-7.

**Conclusions:** The importance of this study is that we used a chronic large urethral defect animal model and clearly found that cell-seeded transplants were superior to nonseeded. In addition, bladder washing was a feasible method for harvesting viable autologous cells in a noninvasive way. There is a place for considering tissue-engineered transplants in the surgical armamentarium for treating complex urethral defects and hypospadias cases.

## Introduction

Hypospadias is a common congenital malformation caused by a default in the normal penis development, resulting in a defect of the ventral urethra, with the urethral meatus located below its normal position, usually associated with a deficiency of the ventral prepuce and an abnormal penile curvature (1). It occurs approximately in 1 in 150 to 300 live births (1–3).

Hypospadias treatment is surgical. Mild case (distal hypospadias) repair is usually successful with numerous techniques, but severe case (proximal hypospadias) treatment may be challenging due to the lack of healthy tissue for the urethral reconstruction. In these cases, multiple urethral tissue substitutes have, so far, been described for creating a neourethra, such as the inner prepuce, buccal mucosa, bladder mucosa, postauricular grafts, among others (4–9). These substitutes have well documented side effects such as donor site morbidity and present mechanical and biological differences compared with the native urethra, and sometimes may not even be available due to multiple previous surgical interventions.

In theory, urethral tissue engineering could be a solution to problems related to traditional urethral substitutes, by offering an off-the-shelf neourethra with the same properties of the native urethra. Neourethras had been created by using two main strategies involving scaffolds (10–12). In one strategy, acellular scaffolds, either of natural origin or synthetic (or a hybrid), have been used in both animal and clinical studies with favorable results for the correction of small defects surrounded by a good vascular bed (13–17). In a second strategy for neourethra regeneration, cell-seeded scaffolds, created by seeding autologous cells on scaffolds, have demonstrated superior results for the correction of bigger defects compared with acellular scaffolds and demonstrated increased vascularization and decreased inflammation and fibrosis (12, 18–20).

There are several cell types used for urethral reconstruction, the most common are autologous urothelial cells (21), buccal mucosa cells (22), keratinocytes (23), fibroblasts (24), and smooth muscle cells (25). These cells can be obtained by biopsy of the tissue, or as in case for urothelium, by a non-invasive method such as bladder washes (26–28). In a clinical study comprising six patients with severe hypospadias, bladder washing was used as the source for autologous urothelial cell harvesting (29, 30).

However, the application of tissue-engineered neourethras in clinical practice is still scarce. Translation to the clinic is limited by the lack of an ideal neourethra and the complex methods involved in its development (11, 12, 31). Simplification of its creation processes could allow its wide application in patients.

Our aim was to create a straightforward tissue-engineered urethral construct by seeding a porcine small intestine submucosa scaffold with urothelial cells obtained by bladder washes and test it in a chronic large urethral defect in a rabbit model.

## Materials and Methods

### Ethical Considerations

This study was approved by the ethical committee at the Hospital Universitario La Paz (CEBA-08-2016) and the Madrid Community Environmental Concierge (PROEX-186/16).

### Creation of the Chronic Large Urethral Defect Model

**Figure 1:**
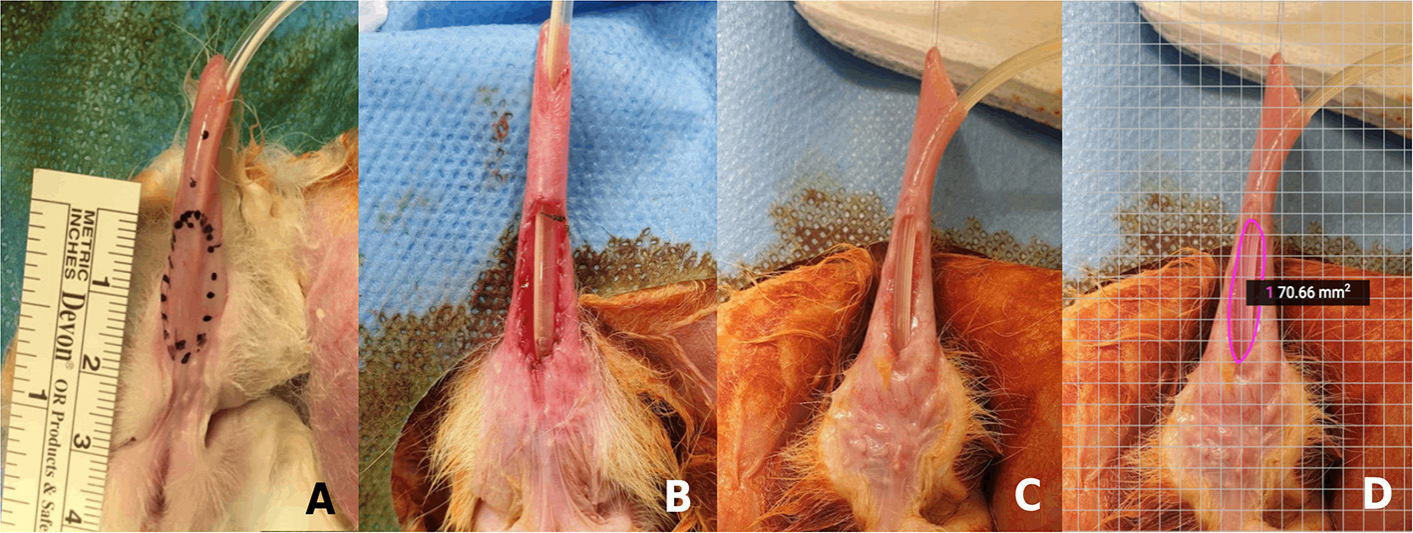
Chronic large urethral defect model: **(A)** Rabbit penis with the drawing of the area of urethral resection to create the defect. **(B)** Large urethral defect newly created. **(C)** Chronic large urethral defect after 3 months. **(D)** Calculated area of the urethral defect (SketchAndCalc^TM^ application).

Twenty-two adult (16 weeks old) giant New Zealand rabbits (*Oryctolagus cuniculus*), 4.5 (4, 5) kg of weight, were operated under general anesthesia to create a chronic urethral defect simulating the large urethral defects of proximal hypospadias. An elliptic segment of approximately 70 mm^2^ (longest 18 mm and widest 5 mm) was resected in the ventral urethra, including the urethral mucosa, subcutaneous tissue, and skin, preserving the glans (the glans was left intact in the model to avoid the need of a urethral catheter due to edema and risk of acute urinary retention after the urethral repair). The edges of the defect were sutured to join the skin with the urethral mucosa to create a stable elliptic defect (Figure 1). The wellbeing of the subjects was evaluated with a specific animal supervision protocol. A welfare scale of 0–12 points was used, where 0 points corresponded to a normal status and 12 points being the endpoint criteria that required early termination of the animal according to principles for animal ethics (Table 1).

**Table 1:** Rabbit welfare scale applied for animal supervision.

**Rabbit welfare scale**		
**Parameters**	**Frequency**	**Score (points)**
Weight	72 h	Loss of 10% = 1; 20% = 2; 20% = 3
Secretions	24 h	0–1–2–3
Fur	24 h	0–1–2–3
Automutilation	24 h	12
Surgical wound	24 h	Well 0; swollen 1; suppurating 2
Shrieking	24 h	0–1–2
Concealment posture	24 h	0–1–2

After 5–6 weeks, the model reproducibility and stability over time were studied, by macroscopic evaluation of the presence of inflammation and infection in the penis and measuring the area of the urethral defect. This area was accurately calculated by using the SketchAndCalc^TM^ application, which assessed the surface by using a photo with a reference scale. In two of the rabbits, the model was left intact to evaluate the long-term stability after 3 months. In this group, the area of the defect was remeasured with the application, and voiding cytourethrogram and histological analysis were performed.

### Creation of the Tissue-Engineered Urethra

Urethral tissues were created with autologous urothelial cells, obtained with a noninvasive method as described before (26–28). In brief, autologous cells were harvested from bladder washes, under general anesthesia, just before performing the surgery to create the urethral defect. To achieve the bladder washes, the urethra was catheterized with an 8 Ch Foley catheter, urine in the bladder was extracted, and several bladder lavages were performed by introducing 50 ml of physiological saline in the bladder and extracting the same volume until collecting a total of 300 ml of fluid (six to eight times). Bladder wash fluid was centrifuged at 1,500 rpm for 10 min, washed twice using Dulbecco's modified Eagle's medium (DMEM). Thereafter, the pellet was resuspended in 3 ml of CnT-Prime® culture medium (Cell-n-Tech, Bern, Switzerland) supplemented with fetal bovine serum (Fisher Scientific S.L., Madrid, Spain) and antibiotics (Penicillin–Streptomycin Mixture 5,000U/5,000 μg Lonza, Barcelona, Spain Barcelona, Spain). The final cell suspension was cultured in a 10-cm^2^ cell culture well-coated with a Human Recombinant Laminin Mixture (Biolaminin 521 LN 5 μg/ml and Biolaminin 511 LN 5 μg/ml, Biolamina AB, Sundbyberg, Sweden). At subconfluence, (70–80% of confluence), the cells were detached using accutase (CnT-Accutase-100, Cell-n-Tech, Bern, Switzerland) and seeded in a scaffold of decellularized porcine small intestinal submucosa matrix (SIS matrix, Biodesign® 1-layer tissue graft, Cook Biotech Europe APS Bjaeverskov, Denmark) with CnT-Prime® culture medium without FBS. After 24 h, the tissue constructs were transferred into an air–liquid interface using Transwell inserts (Falcon® Permeable Supports for 6-well Plate with 1.0 μm Transparent PET Membrane, Corning Optical Communications, S.L.U, Madrid, Spain) in order to stimulate the stratification of the epithelium and thereafter cultivated for 3 weeks.

The constructs created had a rectangular shape of approximately 30 × 7 mm, according to the size of the SIS matrix used. A small piece of each construct (10 × 7 mm) was used for histology and immunoassay, and the remaining (20 × 7 mm) segment of the constructs was used to perform reconstructive urethroplasty in the hypospadias model.

### Urethral Defect Repair

The 22 rabbits were divided into three groups (Table 2): Group-A or control group (two rabbits), in which no reconstructive urethroplasty was performed, and the model was left intact; Group-B or SIS matrix urethroplasty group (10 rabbits), in whom a reconstructive urethroplasty was performed with the porcine small intestine submucosa matrix (Biodesign® 1-layer tissue graft, Cook Biotech Europe APS Bjaeverskov, Denmark) without cells; Group-C or urethral tissue urethroplasty group (10 rabbits), in which a reconstructive urethroplasty was performed with the urethral tissue constructs created by seeding the SIS matrix with autologous urothelial cells.

**Table 2:** Evaluation of urethral tissues in the animal model.

**Group**	**Description**	**Animals (*n*)**
(A) Control	No reconstructive urethroplasty was performed, and the hypospadias model was left intact	2
(B) SIS matrix urethroplasty	Reconstructive urethroplasty was performed with the porcine small intestine submucosa matrix (Biodesign® 1-layer tissue graft, Cook-Biotech®) without cells	10
(C) Urethral constructs urethroplasty	Reconstructive urethroplasty was performed with the urethral tissue created by seeding the small intestinal submucosa matrix (SIS) matrix with autologous urothelial cells.	10

Urethroplasty pair was performed approximately 5–6 weeks after the urethral defect model creation to ensure good healing and to allow cell culture to be carried out. Prophylactic antibiotic was administered using ceftriaxone (20 mg/kg) before the procedure. During the reconstruction, an 8 Ch urethral catheter was placed to the bladder, and the penile skin was mobilized. The material chosen for urethroplasty according to the treatment group (SIS matrix alone or urethral tissue) had dimensions of approximately 20 mm × 7 mm, corresponding approximately to the size of the urethral defect. The material was implanted in a “on-lay” mode, placing it on the ventral side of the hypospadias defect to complete the urethral cylinder. Fixation of the material was performed with a 6-0 poliglecaprone absorbable monofilament suture (Monocryl®). The urethroplasty was covered with the penile skin adjacent, sutured in the ventral midline, avoiding suture overlapping. The urethral catheter was removed at the end of the procedure, due to the tendency of rabbits to bite and extract the catheters. Intramuscular analgesia with Meloxicam (1 mg/kg) was administered every 24 h for 2 days. The welfare scale specific to supervise the rabbits was applied during the postoperative period.

The results of the urethroplasty were evaluated 4 weeks after the reconstructive surgery. The rabbits were examined under general anesthesia. Macroscopic examination of the penis was done by a blinded external evaluator to determine if the correction of the urethral defect was achieved, to assess the existence of curvatures of the penis and to evaluate the aesthetic appearance, using subjective aesthetic scale of 0–10 points (0 being the worst appearance, 10 the best appearance). Photographs were taken to measure the area of the urethral defect in cases of urethral fistula o dehiscence, and this area was calculated using the app SketchAndCalc^TM^. Voiding cystourethrogram was also performed by filling the bladder with iodinated contrast, to determine and document the caliber of the urethra and the presence of strictures or fistulas. After full bladder filling, animals presented with penile erection, which allowed to confirm the absence of penile curvatures. Afterward, termination of the animals, and histological and immunohistochemical examinations were carried out.

### Histology and Immunohistochemistry

The urethral tissue constructs and the rabbit penises were fixed in buffered formalin, dehydrated in ascending series of ethanol, and finally embedded in paraffin. Transversal sections of 7 μm of the urethral tissues and axial sections of 5 μm of rabbit penises were processed and stained with hematoxylin and eosin (H&E). The presence of urothelial cells and epithelial stratification were evaluated. Urothelial cells were characterized using reacting monoclonal mouse anti-cytokeratin 7 antibody (CK7 Clone OV-TL 12/30; Dako Omnis®, Dako, Glostrup, Denmark). Immunolabeling was performed using the horseradish peroxidase (HRP) detection system, visualized with 3,3′-diaminobenzidine (DAB) Chromogen (EnVision FLEX+ mouse, high pH K8002 secondary antibody; Dako Omnis®), and sections were counterstained with hematoxylin. In total, two slides of H and E and two of CK7 per each tissue construct and penis were analyzed.

### Statistical Analysis

Numeric data were analyzed using SPSS (SPSS Inc., Chicago, IL, USA). Outcomes of group A, B, and C were compared. Categorical data were compared using Chi-squared test. Continuous data were presented using median and range and compared using Mann–Whitney *U*-test. Normally distributed continuous data were presented as median and standard deviation. Differences were considered statistically significant at *p*-values < 0.05.

## Results

### Chronic Large Urethral Defect Model

All subjects survived the intervention. All presented with mild hematuria, self-limited in the first postoperative hours and minimal discomfort [1 (0–2) point in the welfare scale]. No change in weight was observed. There were no signs of inflammation or infection in the surgical area, or injuries in the adjacent tissues. In all rabbits, the urethral defect was maintained without presenting reclosure. The mean area of the defect at 5 weeks was 70.3 ± 2.5 mm^2^. In the two rabbits of group-A, in which the model was left intact, there were no changes in the created defect, neither in its size (*p* = 0.35), nor in the characteristics of the tissues at 3 months after surgery (Figure 1C). In the cystourethrogram, a large urinary leak was identified at the site of defect, with a normal posterior urethra and without signs of strictures. In the histological study with H and E, it was found a wide hypospadias-like defect on the ventral aspect of the penis, with a keratinized stratified epithelium on the edges of the defect and a urothelial-type epithelium on the dorsal aspect and lateral faces of the native urethra, with a transition to the keratinized epithelium next to the edges of the defect. No signs of inflammation were evident. The vascularization of the edges of the defect was adequate, and there was no significant fibrosis.

### Establishment of the Tissue-Engineered Urethra

Urothelial cultures were established in all rabbits by the bladder washing method. In 7 of the 22 rabbits (31.82%), it was necessary to repeat the washing once, since no colonies were formed within the first 14 days. The average of washes was 1.32 (±0.48) per rabbit. No more than two washes were necessary in any case. The final cell suspension from the bladder washing contained a mixture of different cell types (red blood cells, white blood cells, urothelial cells, among others). A volume of 2 ml of cell suspension was seeded in the laminin-coated wells, achieving a good adhesion of the urothelial cells to the well. The remaining cells were washed out with the medium changes.

**Figure 2:**
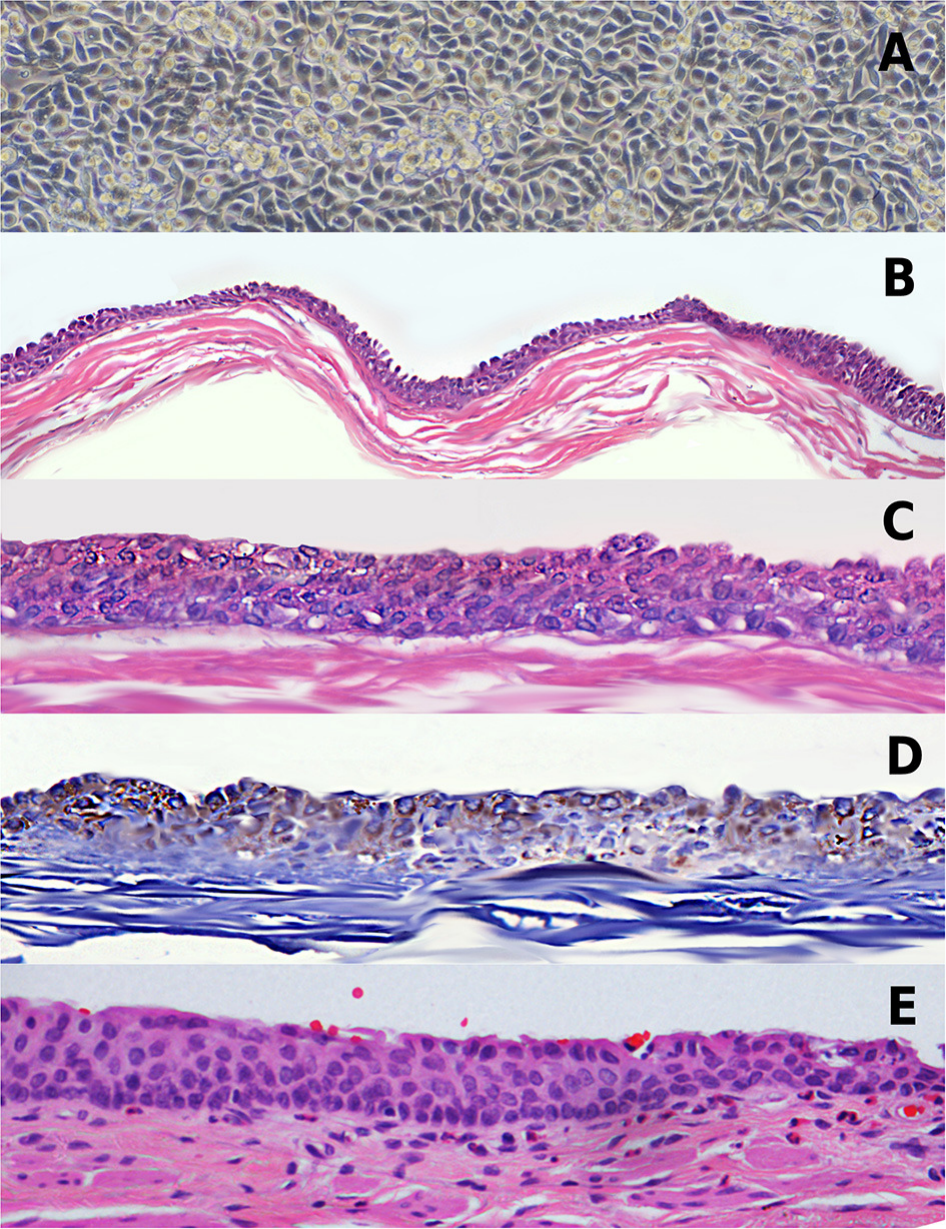
Tissue-engineered urethra. **(A)** Urothelial cell culture at confluence. **(B)** Hematoxylin and eosin (H&E) stain of the construct showing a stratified urothelium on the small intestinal submucosa matrix (SIS) matrix, × 4 magnification. **(C)** Same as **(B)** in × 10 magnification. **(D)** Immunoassay with urothelial cell marker CK7. **(E)** Example of native rabbit penile urethra.

The first urothelial cell colonies appeared at a median of 8 (4–14) days after seeding. The cultures reached cell subconfluence (Figure 2A) at a median of 15 (11–21) days. The cells were passed and seeded on the SIS matrix with good adhesion to the matrix in all 100% samples. The culture was carried out in an air–liquid interface for a median of 21 (18–23) days and developed stratification of the epithelium in all cases (Figures 2B,C).

In all cases, a stratified epithelium of three to five cell layers was found similar to the epithelium of the native rabbit urethra (Figure 2E), without metaplasia or cell atypia (Figure 2C). The immunoassay study demonstrated CK7-stained cells (Figure 2D).

### Urethroplasty

**Figure 3:**
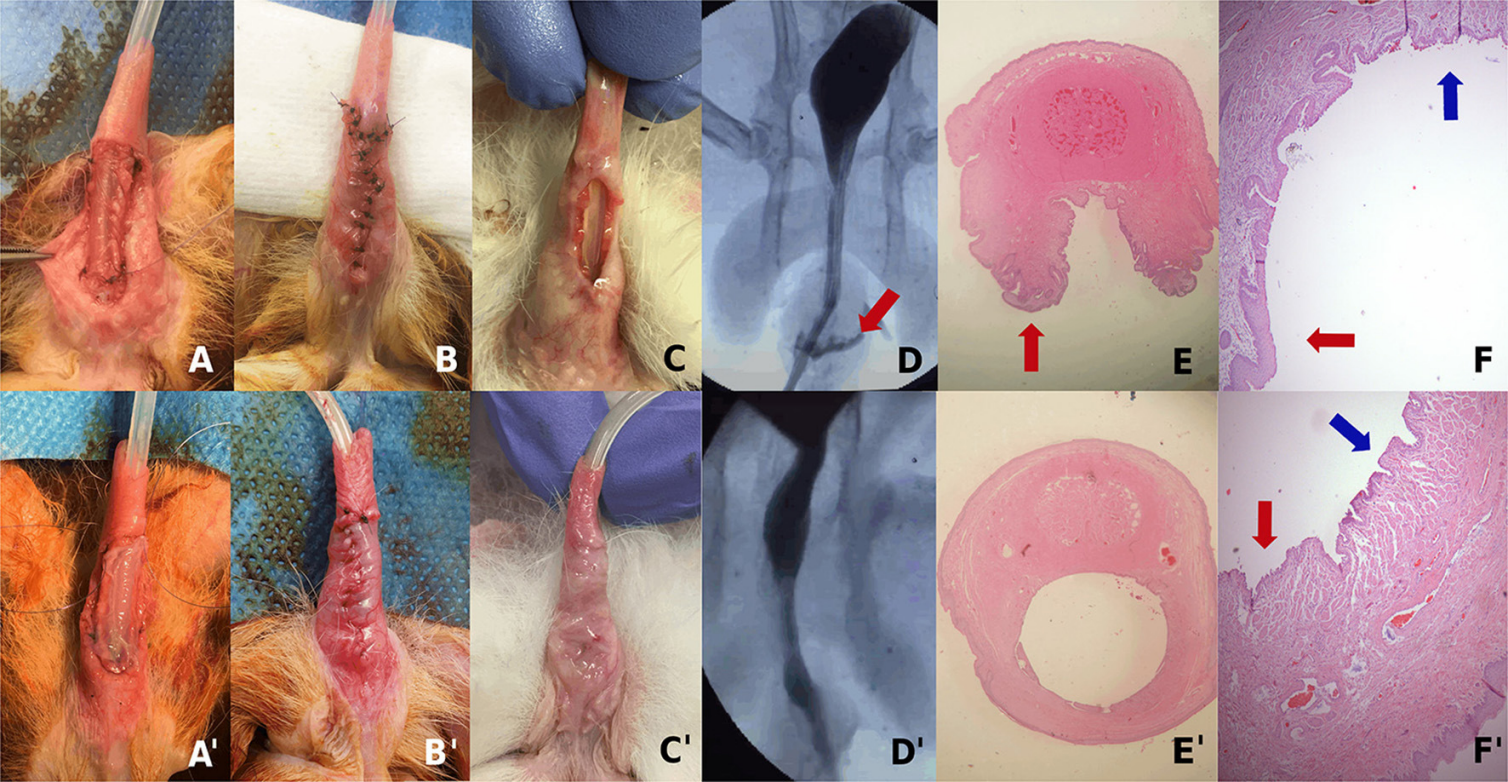
Examples of a rabbit of group-B **(A–F)** and group-C **(A′–F′)**: **(A)** Urethral reconstruction by covering the urethral defect with the SIS matrix. **(A′)** Urethral reconstruction by covering the urethral defect with the tissue-engineered urethra. **(B,B′)** Immediate postop appearance of the urethroplasty. **(C)** Dehiscence of the urethroplasty 4 weeks after the surgery. **(C′)** Result of the urethroplasty 4 weeks after the surgery with complete urethral repair. **(D)** Voiding cystourethrogram showing urethral fistula. **(D′)** Voiding cystourethrogram without fistula. **(E)** Microscopy slide of an axial cut of the penis in the area of urethral defect demonstrating an absence of the ventral urethra. **(E′)** Microscopy slide of an axial cut of the penis in the area of the urethroplasty showing the complete repair of the ventral urethra. **(F)** Magnification of the dorsal urethra (blue arrow) and the continuity with areas of squamous metaplasia (red arrow). **(F′)** Magnification of the ventral neourethra (red arrow) and its continuity with the native urethra (blue arrow).

Urethroplasty was performed in group-B (10 rabbits) and group-C (10 rabbits), after a median of 40 (30–49) days after the first intervention to create the chronic large urethral defect (Figure 3). No urethral repair was performed in group-A (control). The procedure was performed with good tolerance by the subjects, requiring analgesia the first three postoperative days due to minor discomfort (score of 1 point (range 0–3) in the welfare scale). All presented with mild and self-limited hematuria. There were no episodes of urine retention. No animals needed preterm termination.

Surgical manipulation resistance and characteristics were comparable in the SIS matrix only and the TEU. There were no breaks, tears, or deformations in both materials during the procedure. The face of the construct containing the cells was easily identified with magnifying glasses and faced the lumen of the created neourethra.

In the macroscopic examination of the penises 4 weeks after surgery, all rabbits in group-B (10/10) presented dehiscence of the urethroplasty or a large fistula (Figure 3C). In group-C, 5/10 rabbits presented a complete urethral repair without fistulas or stenosis, and 5/10 presented almost total repair but with a small urethral fistula (Figure 3C′). The median calculated area of the urethral defect after urethroplasty in group-B was 61.1 mm^2^ (range 34.0–70.5), and in group-C, it was 0.3 mm^2^ (range 0–12.5) (Table 3), with a statistically significant difference (*p* = 7.85 × 10^−5^). No penile curvatures were identified in any samples. Regarding the aesthetic aspect, this was deficient in group-B, with a score of 2 points (range 1–4) in the subjective aesthetic scale. However, in group-C, the aesthetic result was favorable, with a median score of 8 points (range 5–9) (*p* = 7.85 × 10^−5^). In rabbits of group-A, in which the model was left intact, there were no changes in the created defect, neither in its size nor in the characteristics of the tissues at 3 months after model creation.

**Table 3:** Calculated area of the initial and final urethral defect of all groups using the SketchAndCalc^TM^ application.

**Calculated area of the urethral defect**								
**Group A**			**Group B**			**Group C**		
**Control**			**SIS matrix urethroplasty**			**Urethral construct urethroplasty**		
**Rabbit**	**Initial area**	**Final area**	**Rabbit**	**Initial area**	**Final area**	**Rabbit**	**Initial area**	**Final area**
1	70.66	70.65	1	71.91	48.03	1	72.67	0
2	72.25	72.25	2	67.75	61.15	2	71.81	0
			3	74.07	42.83	3	67.09	0
			4	72.97	33.99	4	70.99	3.24
			5	69.05	64.24	5	68.19	12.51
			6	72.26	70.49	6	67.13	0
			7	68.69	61.14	7	69.36	0
			8	65.10	61.81	8	74.54	0.62
			9	67.62	60.91	9	71.90	1.01
			10	70.19	45.93	10	69.69	10.55
**Median**	71.46	71.45	**Median**	69.62	61.03	**Median**	70.34	0.31
**Mean**	71.46	71.45	**Mean**	69.96	55.05	**Mean**	70.34	2.79
**DS**	1.12	1.13	**DS**	2.82	11.56	**DS**	2.47	4.73

In the radiological evaluation using voiding cystourethrogram, cases with fistula were confirmed. In group-B, 10/10 rabbits presented with urinary fistula (Figure 3D), compared with 5/10 in group-C (Figure 3D′), the difference was statistically significant (*p* = 0.04). No strictures or diverticula were identified in any animals.

In the histological examination, all rabbits in group-B had an absence of the ventral urethra in the areas of uncorrected defect, with continuity between the dorsal urethra and the penile skin and areas of squamous metaplasia in the lateral sites of the urethra (Figures 3E,F). In group-C, rabbits with total correction of the urethral defect had a ventral urethra with multilayered urothelial tissue, and subcutaneous and cutaneous tissue, and in the rabbits with fistula, the ventral urethra had some squamous metaplasia, with the rest of the urethra repaired with normal urothelium (Figures 3E′,F′). Presence of vascular and connective tissues in the repaired urethra areas were identified. No atypia or tumors were recognized in any of the samples. Immunoassay identified CK7-stained cells in the areas of urethral repair.

Supplementary Figures of the urethroplasty results (macroscopic examination, radiology, and histology) of all rabbits are available (Supplementary Figures).

## Discussion

In this study, we can summarize three main findings: we were successful in establishing a reproducible and chronic large urethral defect animal model in rabbits; TEUs were successfully created, using the bladder washing technique as source of cells and SIS matrix as scaffold; and reconstructive urethroplasty using TEUs was superior to the use of acellular scaffolds of SIS matrix in the rabbit model.

In the model of the study, a large ventral urethral defect was created and found stable in the long-term (during 5 weeks in all rabbits and up to 3 months in control rabbits or group-A). In addition, the variation of the size of a urethral defect between animals was minimal, demonstrating the reproducibility of the model. The rabbit model is one of the best animal models for the study of urethroplasty techniques due to its anatomical characteristics (32–34), but there is a lack of an ideal animal model that truly resemble human congenital urethral defects (12).

In human hypospadias, the urethral deficiency is usually associated to a defect of the corpus spongiosum, which gives a poor vascularization in the wound bed (35). Hypospadias animal models usually have plenty of urethral tissue with good vascularization, which could give better results to the experimental studies compared with the clinical practice. For this reason, in this study, we tried to create a hypospadias animal model, with a large urethral defect, to reduce the abundant urethral tissue. None of the animals presented urethral strictures, which demonstrates that the urethral defect was large enough to avoid re-closure of the defect with the adjacent tissue.

Compared with other experimental studies for urethral reconstruction, our model is unique, as the urethral defect in our study was prepared 4–5 weeks before the planned reconstruction to allow settlement of the urethral defect as chronic and simulate the conditions under which urethral repair normally takes place. Previously, in most tissue-engineered urethral experimental studies, the urethral defect has been created intraoperatively at the time of reconstruction (10–12).

The stratified TEUs created in this study had some advantages compared with other types of urethral tissue-engineered constructs previously described (10, 36–38) that make them ideal for translation to clinical setting. The SIS matrix scaffold is a commercially available biomaterial approved for human use since 2004, and its safety in clinical practice has been extensively proven (39–43).

Cell harvesting by the bladder washing procedure has the advantages of low morbidity, avoiding donor site lesions and providing the possibility of repeated procedure if needed (26–30). In the clinical practice, bladder washes could be accomplished in the outpatient clinic in older patients. The type of cell cultures and expansion techniques, without using any feeding cells or any other substance not allowed for human application, is also an advantage of our construct.

The only substance that should be substituted in a human setting is fetal bovine serum (Fisher Scientific S.L., Madrid, Spain) in the cell culture medium, which may be replaced by the patient's serum. Furthermore, with our simple culture techniques, a multilayer construct was accomplished, without requiring expensive bioreactors or complex laboratory machines. Cultures could be elaborated in standard laboratories accredited by Good Manufacturing Practice for human application.

Reconstructive urethroplasty using TEUs was superior to the use of acellular scaffolds in our study. The urethroplasty, using our cell-seeded constructs, allowed complete repair in half of the cases, with only small fistulas in the other half, despite no catheter left in the animals. In a clinical scenario, where urethral catheter may be used, fistula appearance may be further reduced. Also, cell-seeded constructs allowed better healing of the urethral defect, which also improved the cosmetic appearance of the repair. This result is concordant with previous evidence about this topic in preclinical studies, in which cell seeding of tissue-engineered constructs leads to a significant reduction in side effects after urethral repair and has superior results (10–12, 18–20, 37, 38). However, the translation to clinical practice of cell-seeded constructs has been scarce, and interpretation of the results of clinical studies is difficult due to small cohort sizes, short-term results, and most of them being low-evidence studies without randomization or control group (24, 25, 29, 30, 44, 45).

In other animal studies, a full tubularized repair has been described (18, 20), while in clinical scenarios, a common method would be using inlay or onlay procedures (24, 25, 29, 30, 44, 45). It is important to mention that we performed the urethral repair in an onlay fashion to resemble the situation with a poor vascularization as in real human hypospadias, but in the clinical practice, we would recommend using this type of constructs in an inlay fashion, as a two-staged procedure, to enhance optimal vascularization of the construct. We believe that tubularized tissue-engineered constructs would only be an option if a vascular layer or arterial pedicle is associated in the construct; otherwise, the construct would have difficulties to survive in a human scenario with a poorly vascularized wound bed.

Strictures could not be found in any of our specimens. It is possible that the SIS matrix, which is a naturally derived decellularized matrix, reduces the development of fibrosis compared with synthetic materials. In studies with dermal matrices, it has been demonstrated that natural matrices have superior results compared with synthetic matrices, as the latter seem to induce more foreign body reactions including giant cell formation (46).

A limitation of the study was the lack of long-term results. Something that cannot be obtained in a translational manner in an animal study. Further elucidation would need to take place in a human model. Safety of tissue transplants must be proven to ensure the absence of development of atypia or neoplasia. Also, urethral repair stability in a longer period most be proven.

Another limitation is the deficiency of the animal model to really resemble a human hypospadias. The corpus spongiosum is normal in our model, and the glans was preserved. It provided better vascularization compared with the clinical practice, in which most of the theoretical target patients for using tissue-engineered urethral constructs are complex cases with cripple penises and a poor vascular bed.

Finally, the avoidance of using a urethral catheter in our model was due to technical reasons, which could increase the appearance of urine leaks and fistula formation, in all the study groups.

## Conclusions

The chronic large urethral defect model created was harmless to the rabbit, reproducible and stable over time, and may therefore be suitable for use for further development of urethroplasty models and tissue engineering techniques.

Bladder washing was a reliable source of viable urothelial cells for culture. Urothelial cell seeding on an SIS matrix allowed creation of urethral tissues with a stratified epithelium similar to a native urethra and suitable for urethroplasty.

The use of tissue-engineered urethras for urethroplasty in the rabbit model was feasible and presented macroscopic, radiological, and histological superior results compared with the SIS matrix alone.

## Data Availability Statement

The raw data supporting the conclusions of this article will be made available by the authors, without undue reservation.

## Ethics Statement

The animal study was reviewed and approved by the ethical committee at Hospital Universitario La Paz (CEBA-08-2016) and the Madrid Community Environmental Concierge (PROEX-186/16).

## Author Contributions

MA, MF, and PL contributed to conception and design of the study. MA, BS, CC, and MF developed the tissue-engineered urethras. MA, SR, RL, and MM created the hypospadias animal model and performed the urethral repair procedures in the animal model. MA and BS performed the histology and immunohistochemistry. MA, SR, RL, and MM organized the database. MA performed the statistical analysis. MA, MF, and PL wrote the first draft of the manuscript. CC, MM, SR, and RL wrote sections of the manuscript. All authors contributed to manuscript revision, read, and approved the submitted version.

## Conflict of Interest

The authors declare that the research was conducted in the absence of any commercial or financial relationships that could be construed as a potential conflict of interest.
