# Supplementary material for: Creation of Tissue-Engineered Urethras for Large Urethral Defect Repair in a Rabbit Experimental Model
Source: Front Pediatr. 2021 Jun 22;9:691131. doi: 10.3389/fped.2021.691131 (PMC8258112; doi:10.3389/fped.2021.691131)

Supplementary Material

## Supplementary Figures

**Supplementary Figure 1:** Rabbits of group-B (SIS matrix urethroplasty group): (A) Urethral defect before urethral reconstruction with the porcine small intestine submucosa matrix SIS Cook-Biotech®. (B) Immediate postoperative appearance of the urethroplasty. (C) Result of the urethroplasty 4 weeks after the surgery. (D) Voiding cystourethrogram showing urethral fistula. (E) Microscopy slide of an axial cut of the penis in the area of urethral defect demonstrating an absence of the ventral urethra and continuity between the dorsal (open) urethra and the penile skin with areas of squamous metaplasia in lateral sites of the urethra (H&E).

**A B C D E**

1
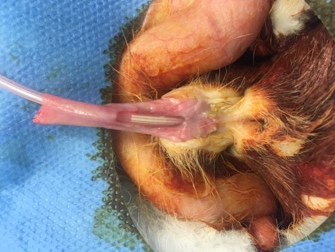

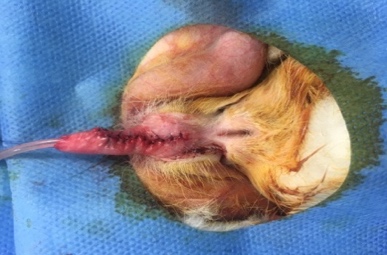

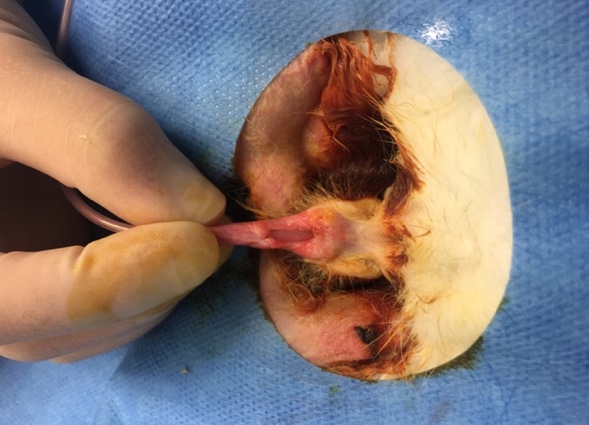

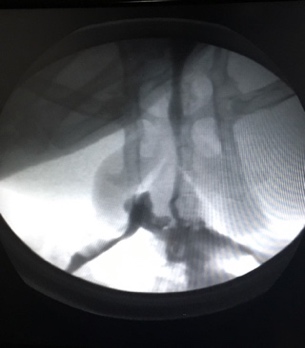

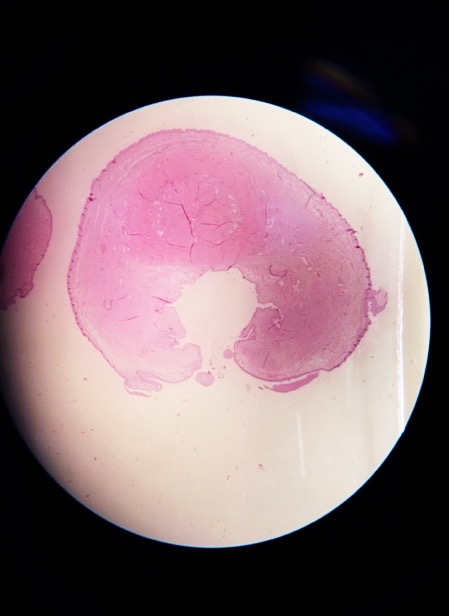


2
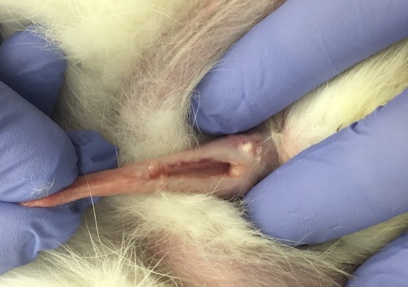

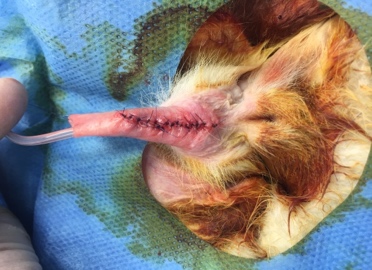

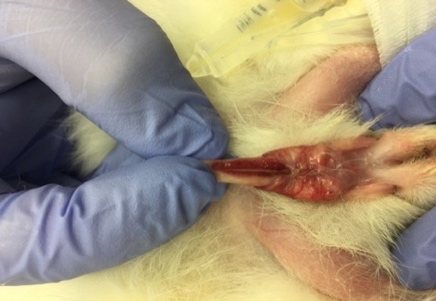

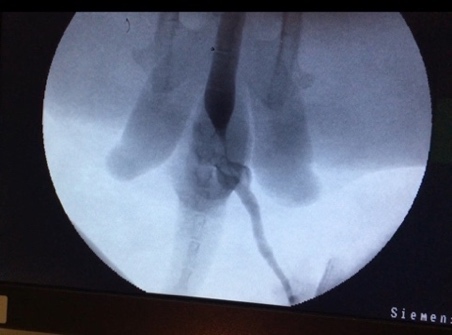

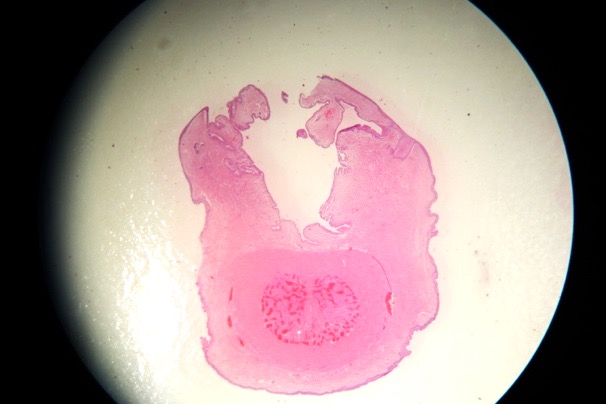


3
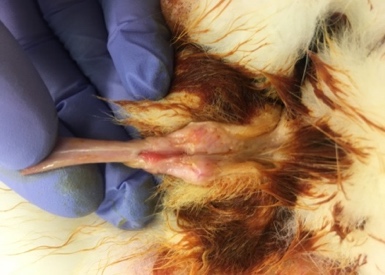

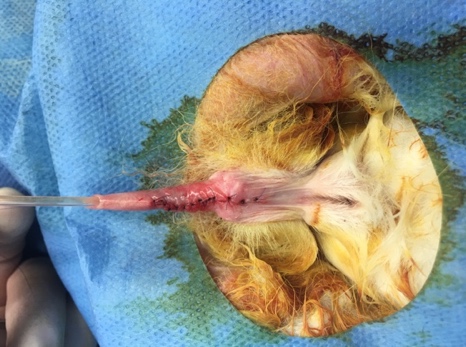

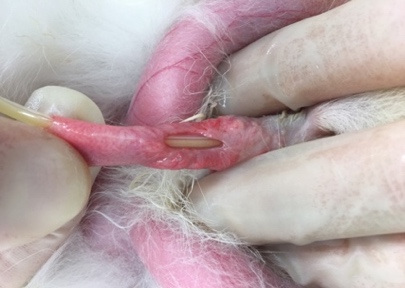

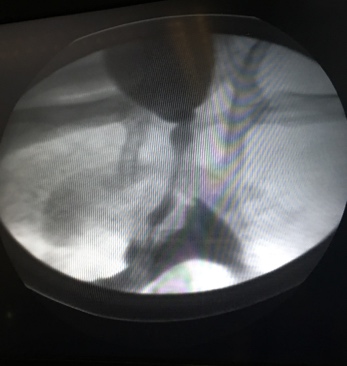

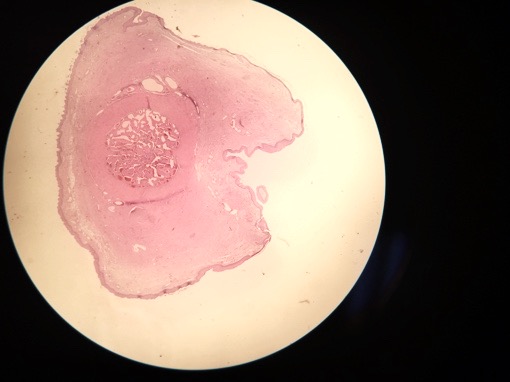


4
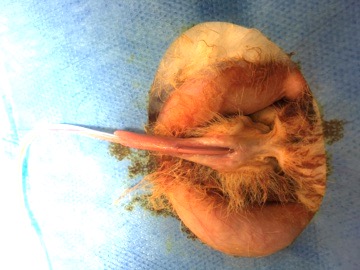

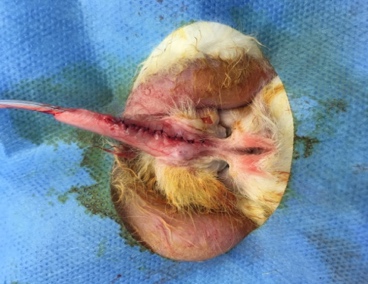

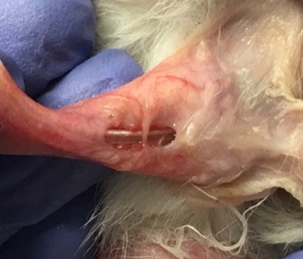

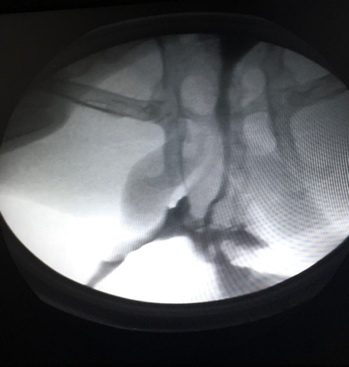

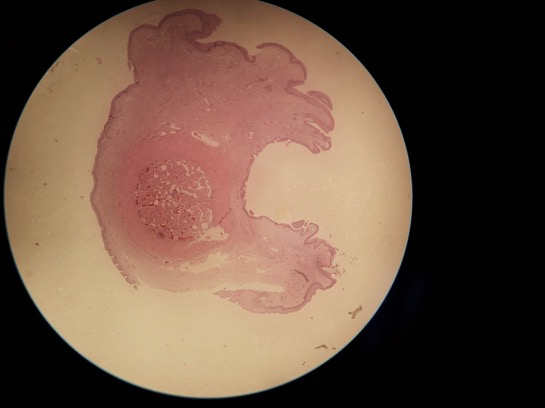


5
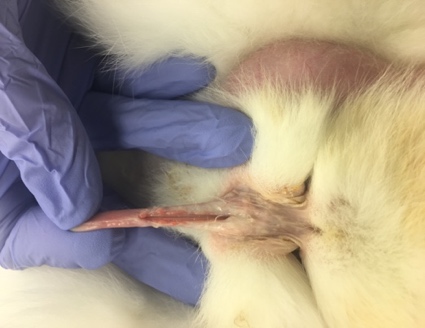

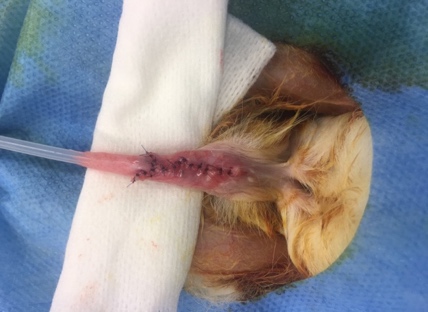

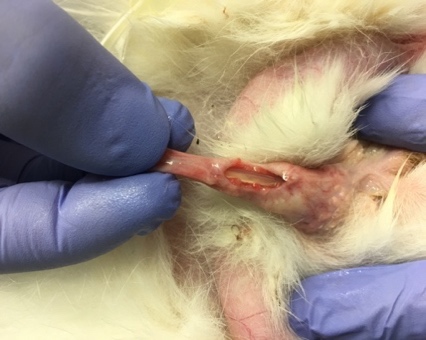

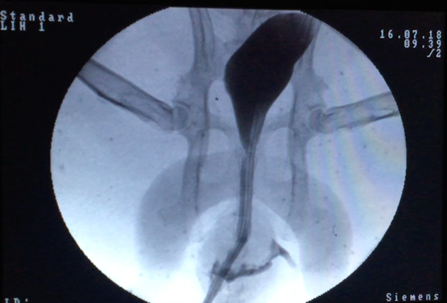

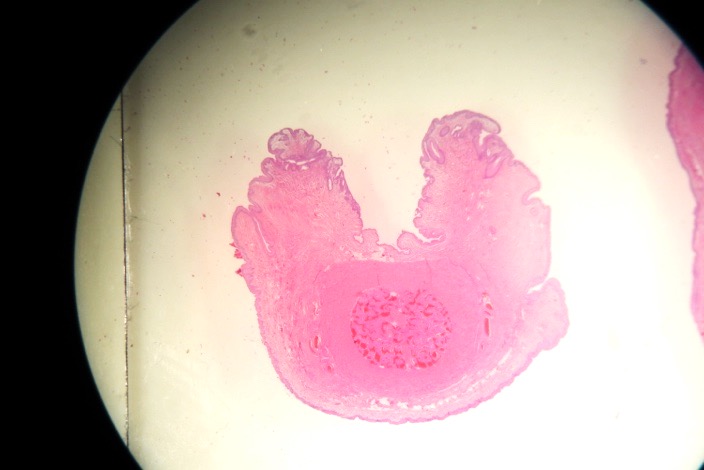


6
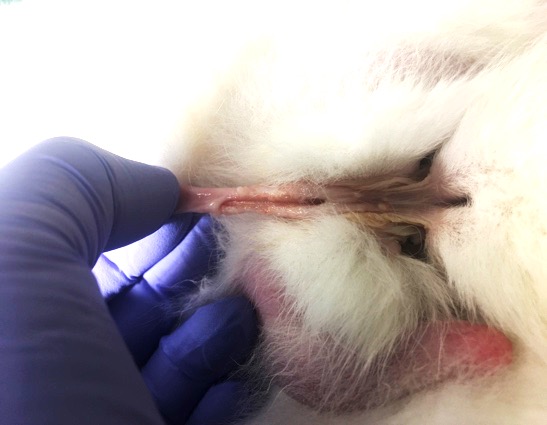

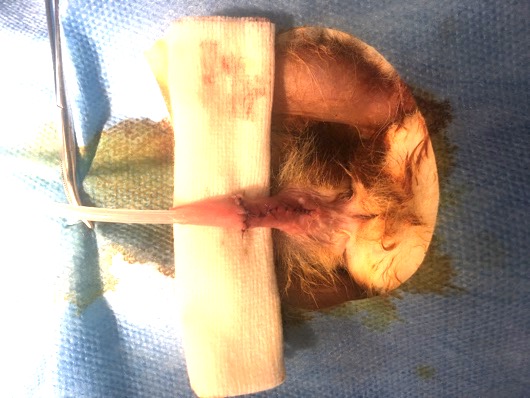

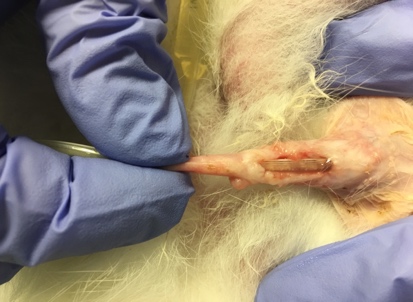

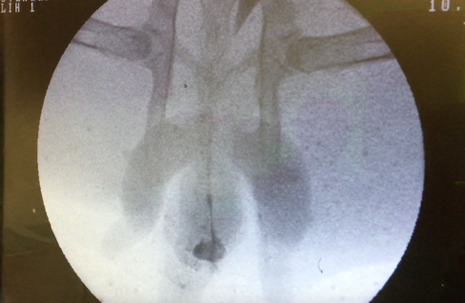

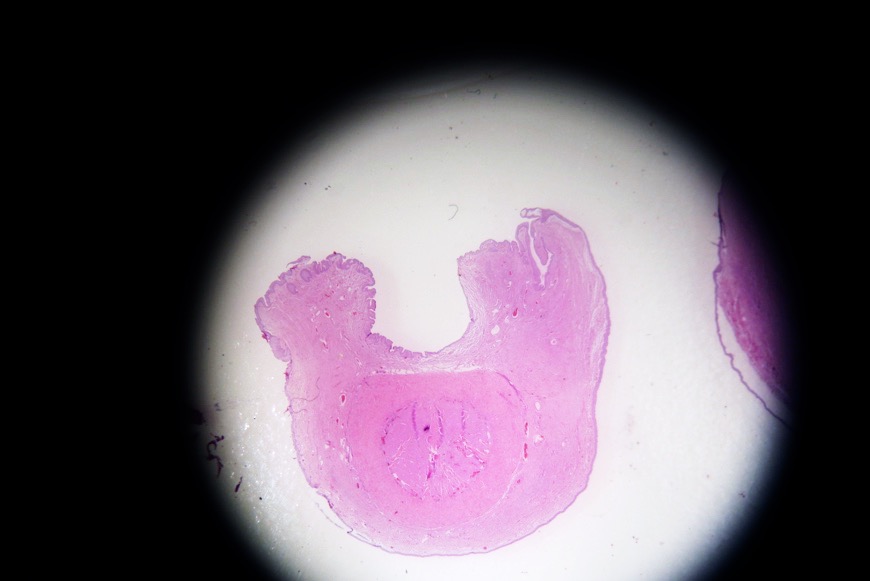


7
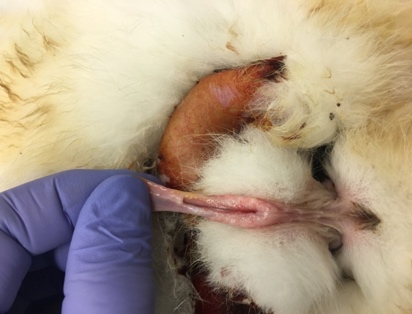

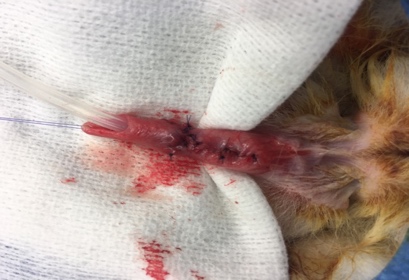

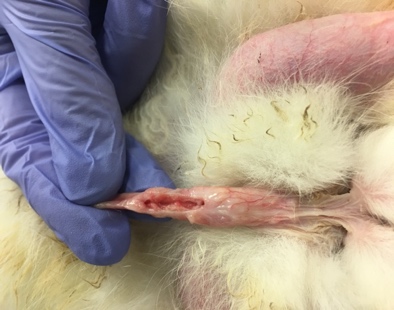

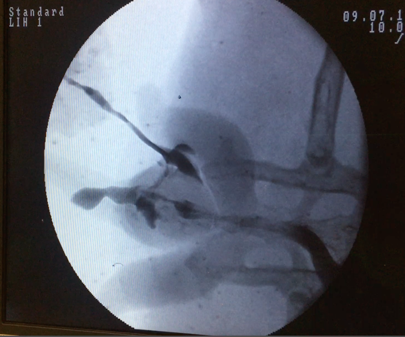

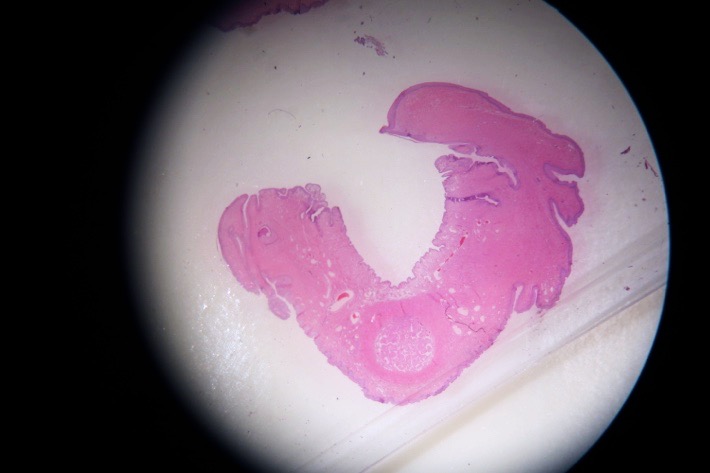


8
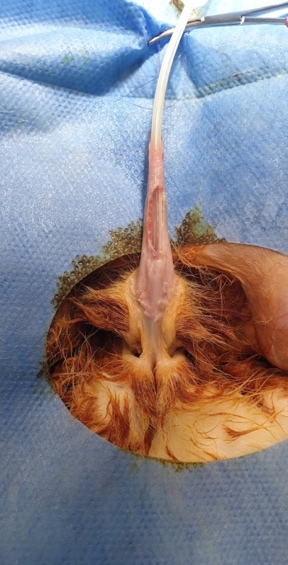

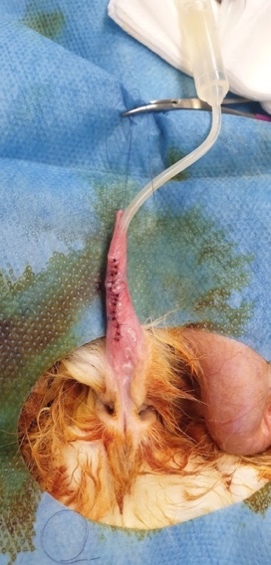

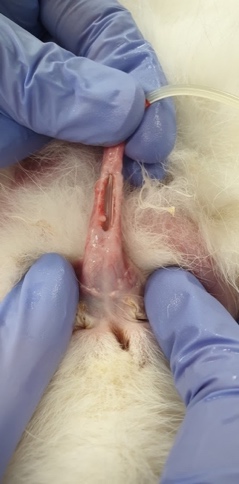

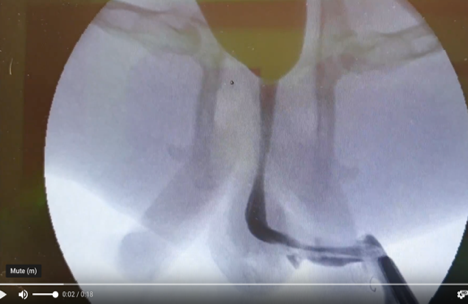

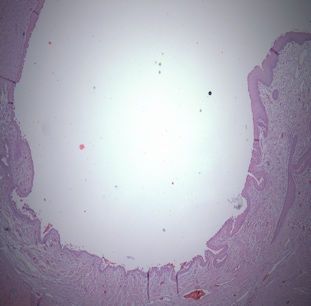


9
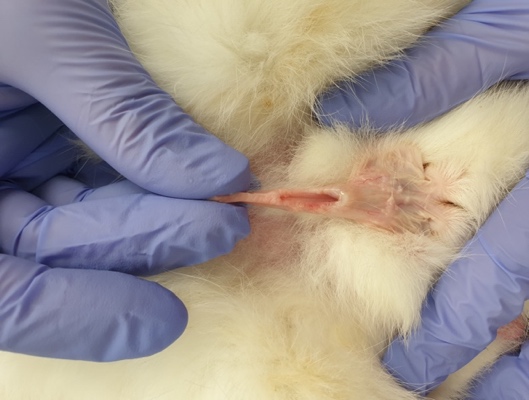

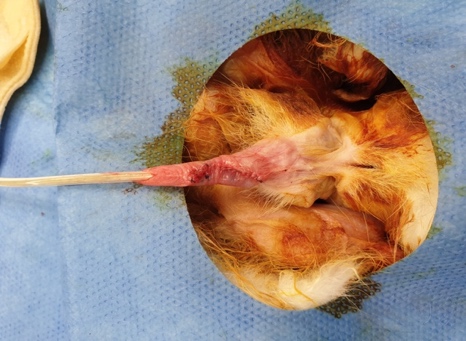

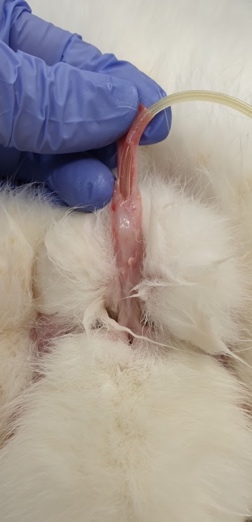

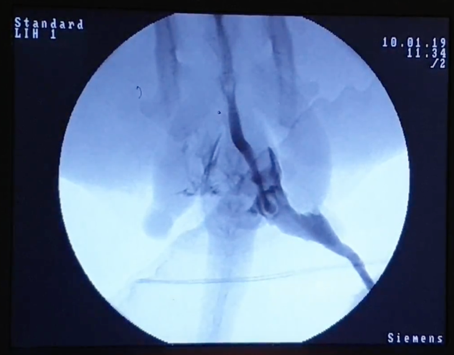

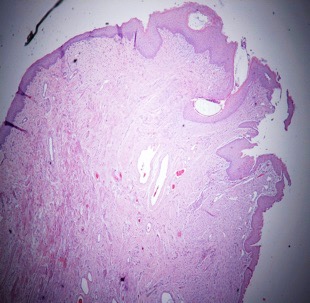


10
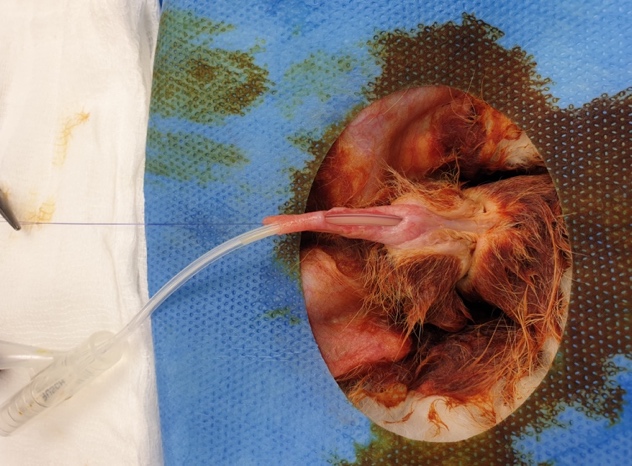

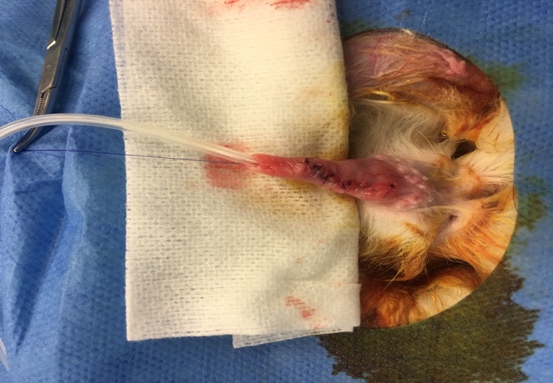

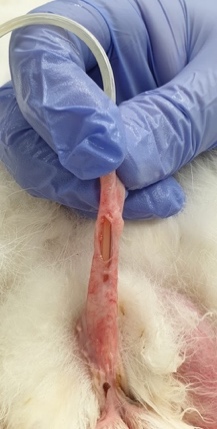

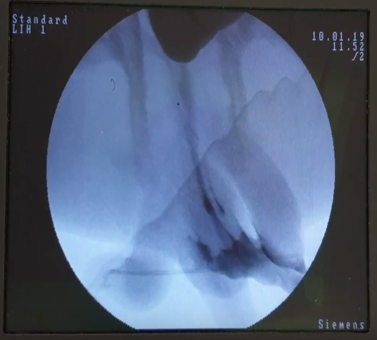

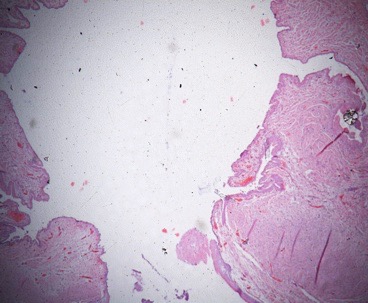


**Supplementary Figure 2: Rabbits of group-C (urethroplasty group with urethral constructs): (A) Urethral defect before reconstruction with the cell-seeded constructs. (B) Immediate postoperative appearance after the urethroplasty. (C) Result of the urethroplasty 4 weeks after the surgery. (D) Voiding cystourethrogram showing urethral fistula or complete urethral repair without fistula. (E) Microscopy slide of an axial cut of the penis in the area of the urethroplasty showing the ventral urethra repaired with multilayer urothelial tissue, or showing urethral fistula with areas of squamous metaplasia in lateral sites of the fistula (H&E).**

**A B C D E**

1
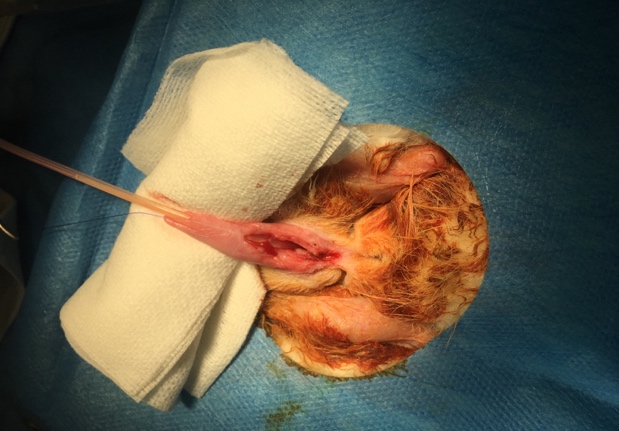

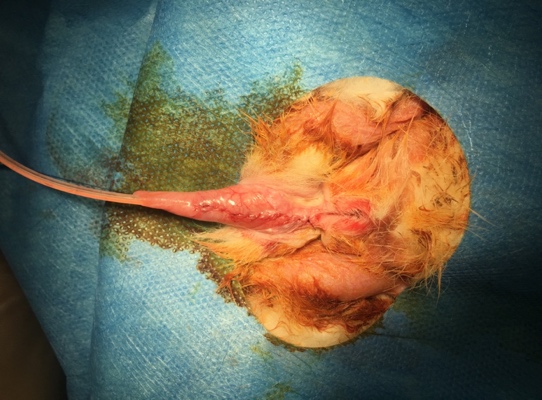

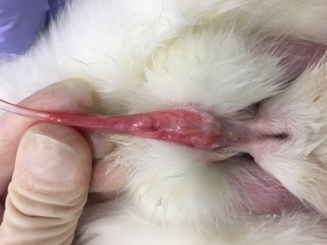

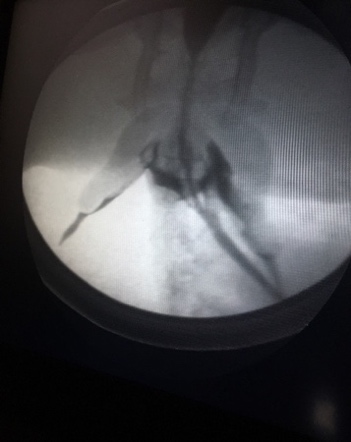

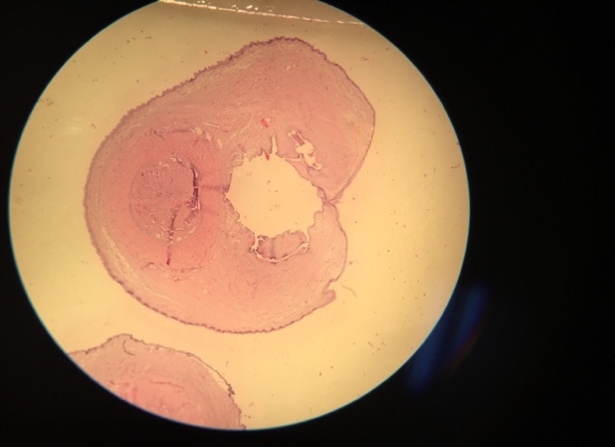


- 2
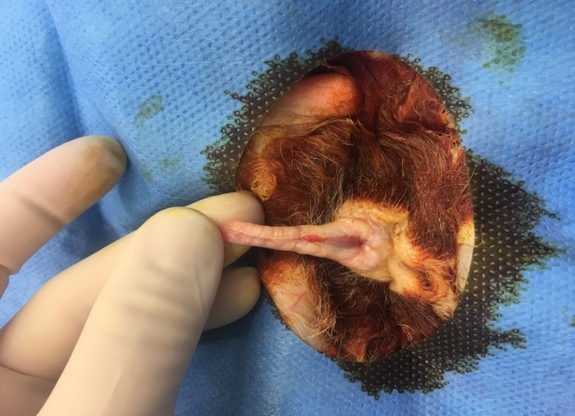

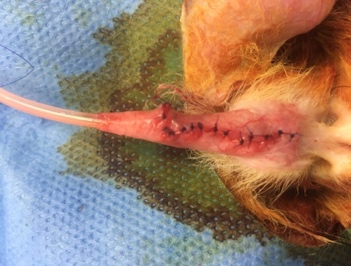

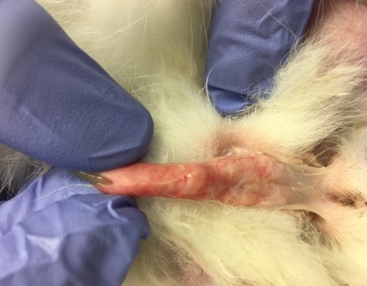

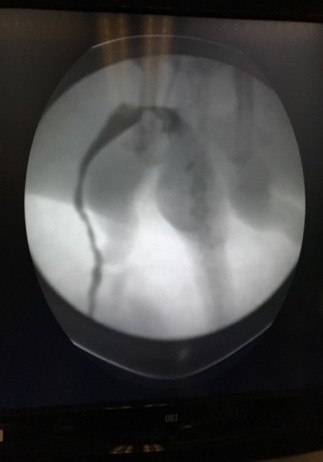

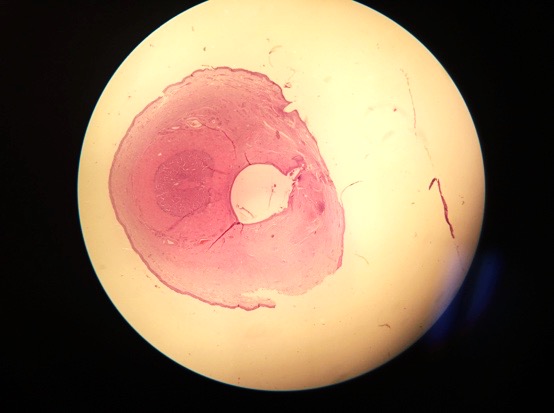


3
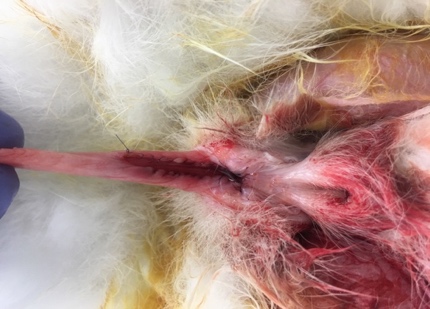

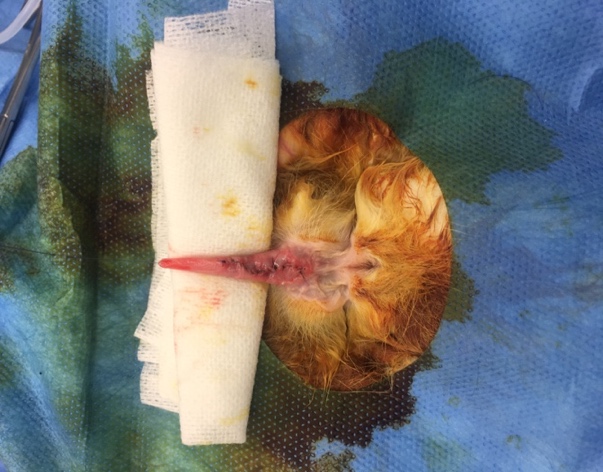

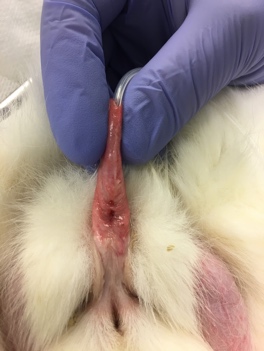

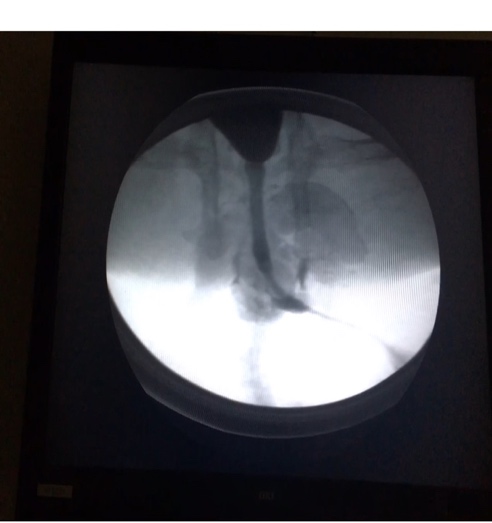

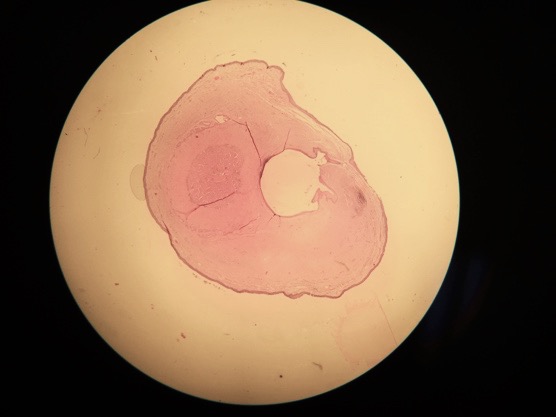


4
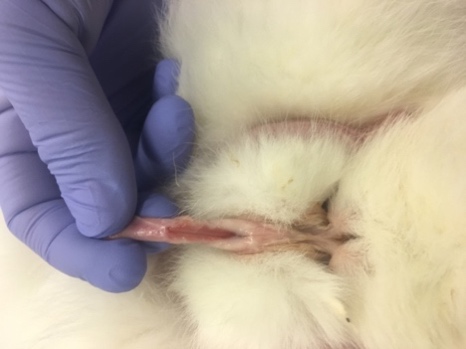

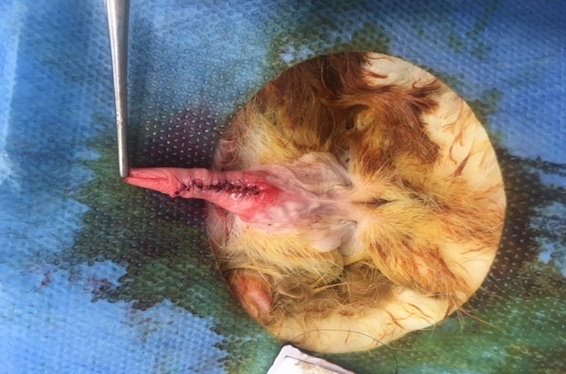

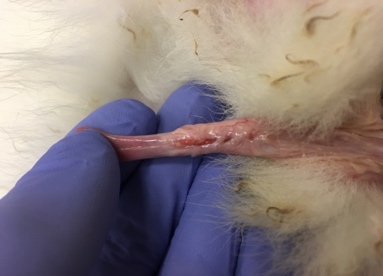

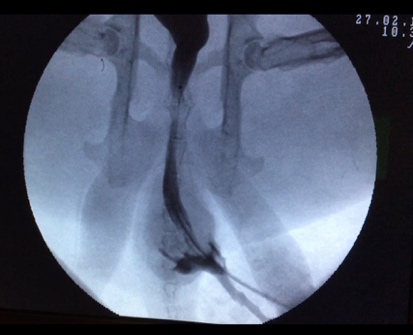

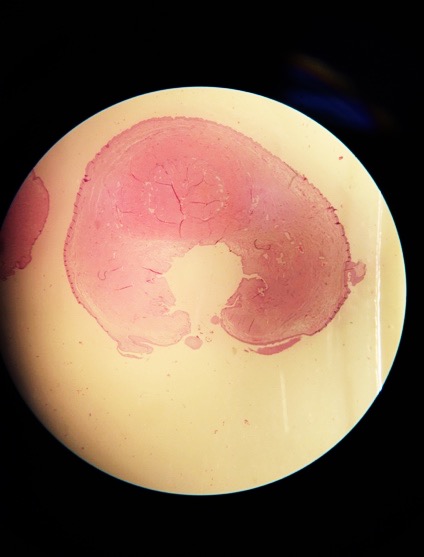


5
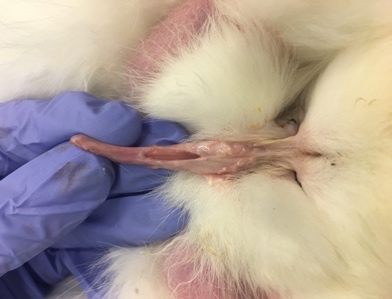

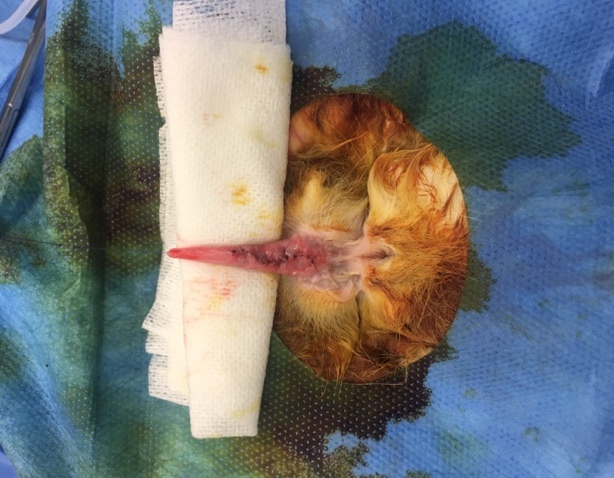

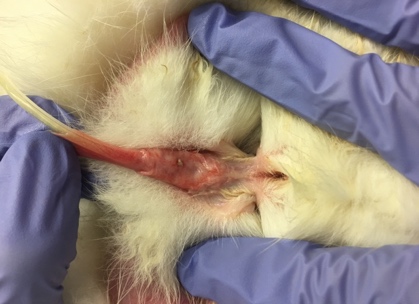

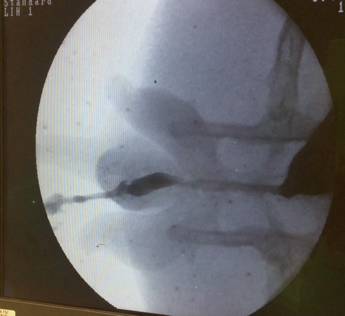

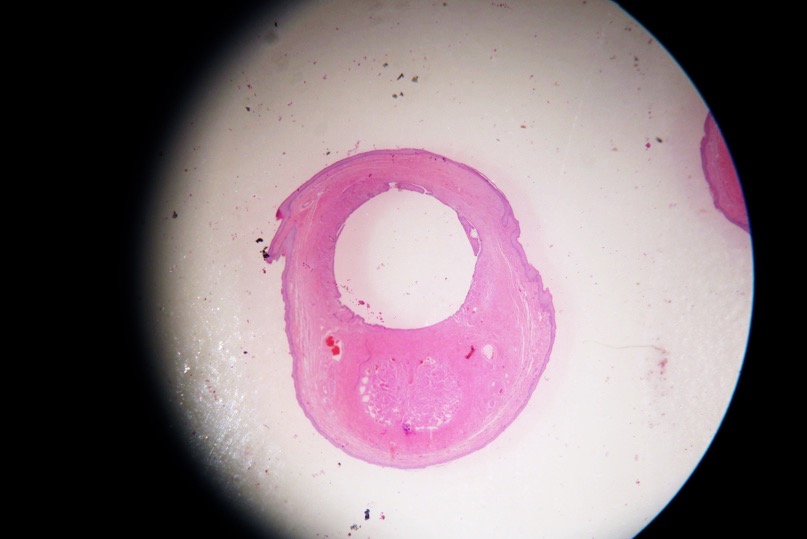


6
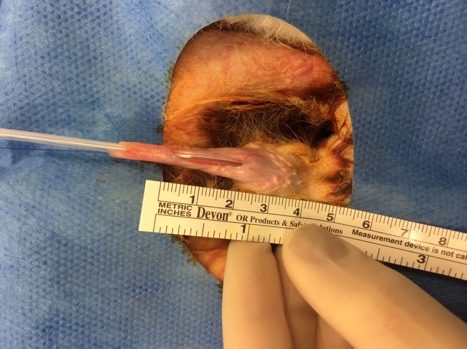

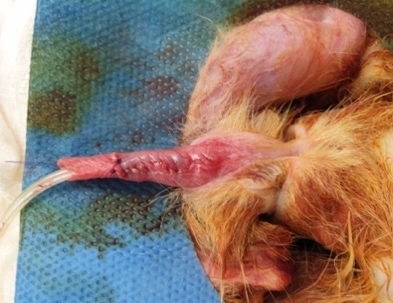

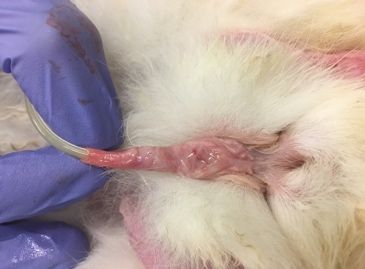

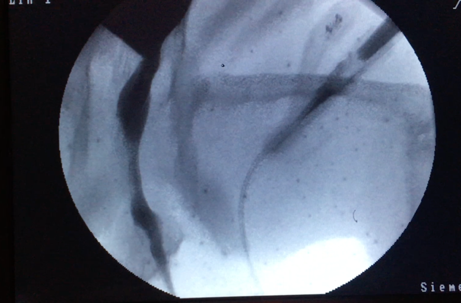

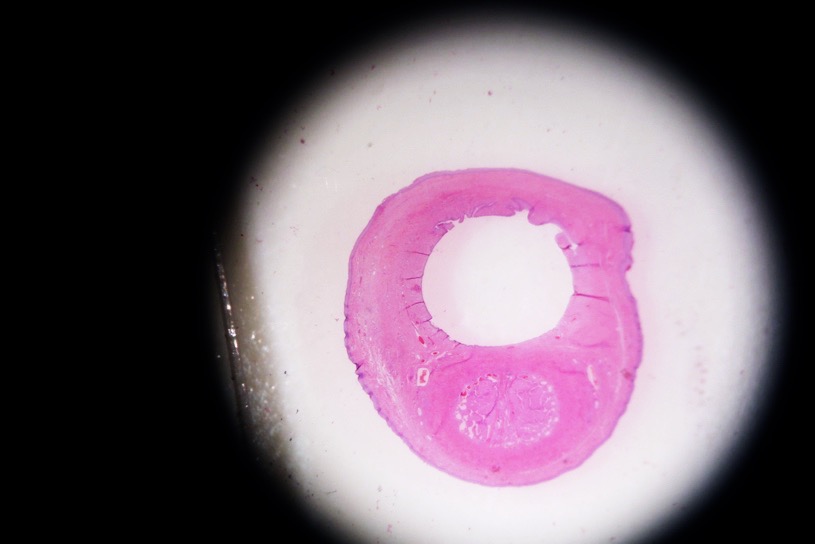


7
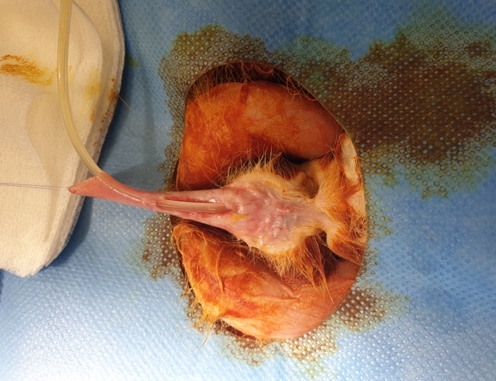

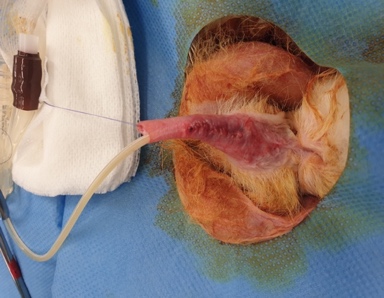

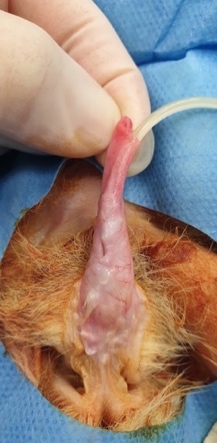

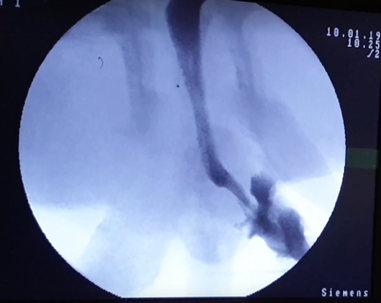

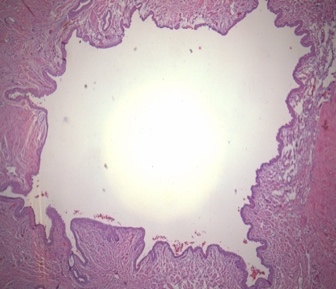


8
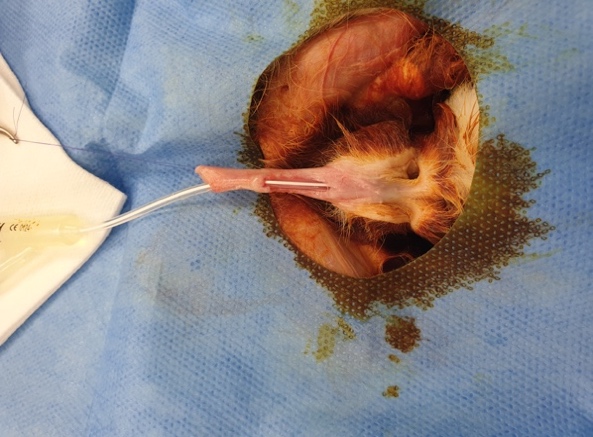

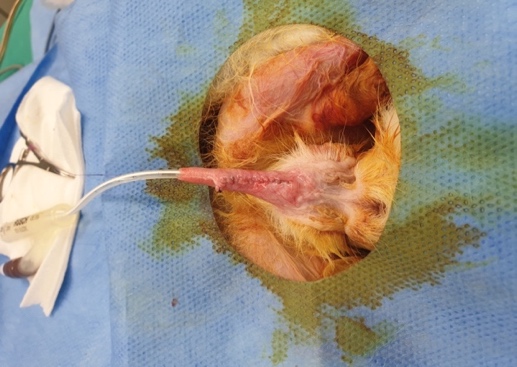

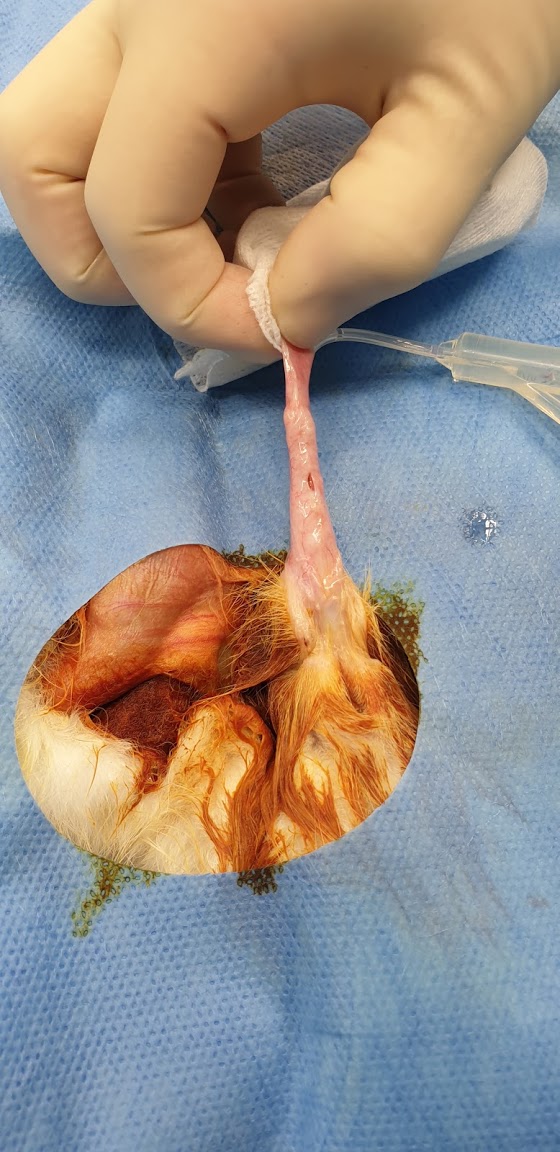

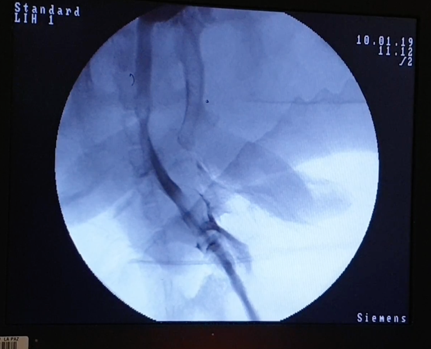

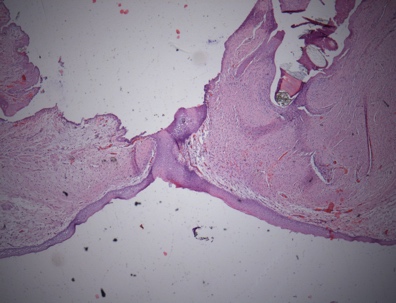


9
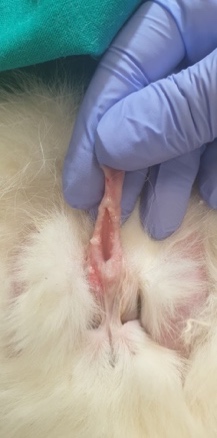

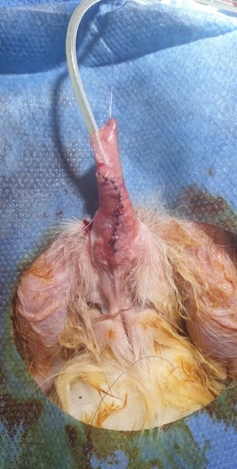

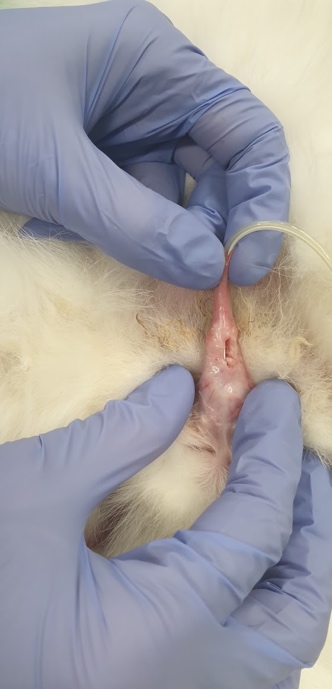

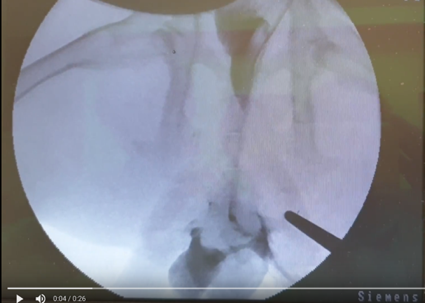

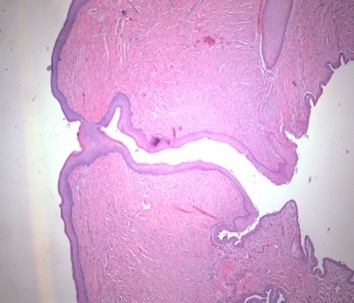


10
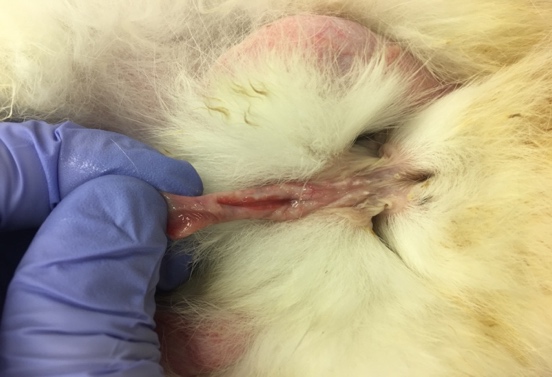

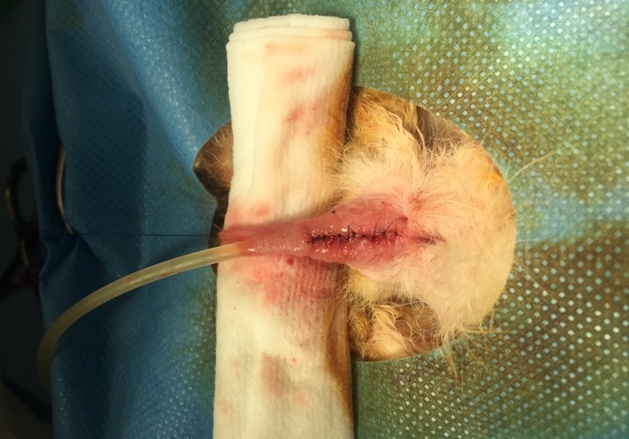

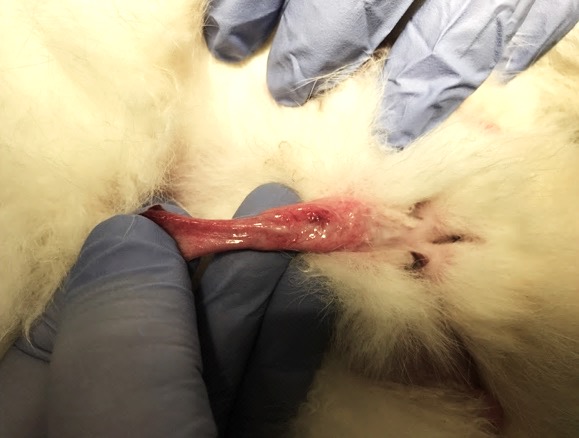

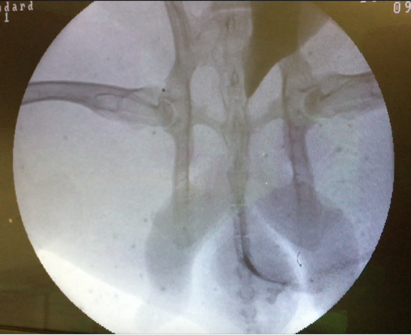

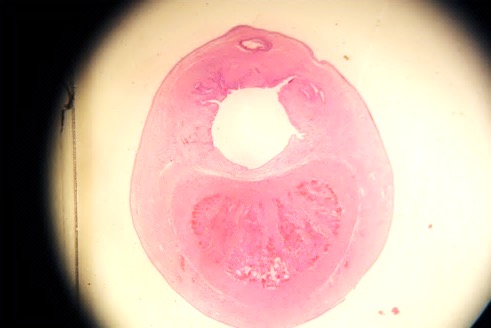

Supplement: Supplementary file 1 [file Data_Sheet_1.docx]
